# Risk Factors Associated with Unsafe Injection Practices at the First Injection Episode among Intravenous Drug Users in France: Results from PrimInject, an Internet Survey

**DOI:** 10.1155/2015/507214

**Published:** 2015-10-05

**Authors:** Anne Guichard, Romain Guignard, France Lert, Elise Roy

**Affiliations:** ^1^French National Institute of Prevention and Health Education, Scientific Affairs Department, 42 boulevard de la Libération, 93203 Saint-Denis Cedex, France; ^2^Faculty of Nursing, Université Laval, Pavillon Ferdinand-Vandry, Bureau 3465, 1050 avenue de la Médecine, Québec City, QC, Canada G1V 0A6; ^3^French National Institute of Health and Medical Research (INSERM), U1018, Hôpital Paul Brousse, 16 avenue Paul Vaillant Couturier, 94807 Villejuif Cedex, France; ^4^Faculty of Medicine and Health Sciences, University of Sherbrooke, Campus de Longueuil, 150 Place Charles-LeMoyne, Bureau 200, Longueuil, QC, Canada J4K 0A8

## Abstract

*Background*. New drug use patterns may increase the risk of human immunodeficiency virus and hepatitis infections. In France, new injection patterns among youths with diverse social backgrounds have emerged, which may explain the persistently high rates of hepatitis C virus infection. This study explores factors associated with injection risk behaviours at first injection among users who began injecting in the post-2000 era. *Methods*. A cross-sectional study was conducted on the Internet from October 2010 to March 2011, through an online questionnaire. Multivariate logistic regression identified the independent correlates of needle sharing and equipment (cooker/cotton filter) sharing. *Results*. Among the 262 respondents (mean age 25 years), 65% were male. Both risk behaviours were positively associated with initiation before 18 years of age (aOR 3.7 CI 95% 1.3–10.6 and aOR 3.0 CI 95% 1.3–7.0) and being injected by another person (aOR 3.1 CI 95% 1.0–9.9 and aOR 3.0 CI 95% 1.3–7.1). Initiation at a party was an independent correlate of equipment sharing (aOR 2.6 95% CI 1.0–6.8). Conclusions. Results suggest a need for innovative harm reduction programmes targeting a variety of settings and populations, including youths and diverse party scenes. Education of current injectors to protect both themselves and those they might initiate into injection is critically important.

## 1. Introduction

Injection drug use has long been regarded as a serious public health issue worldwide. According to a systematic review of the epidemiology of hepatitis B and hepatitis C, approximately 10 million intravenous drug users (IDUs) worldwide are carriers of anti-hepatitis C virus (HCV) antibodies, and 1.2 million IDUs carry HbsAg antibodies [1]. Additionally, approximately 19% of million people who inject drugs may be human immunodeficiency virus- (HIV-) positive [2]. However, the proportion of cases related to drug use varies widely across countries depending on implementation of harm reduction policies, law enforcement practices and drug use practices, and their changes over time. In France, new diagnoses of HIV infection among drug users have dramatically decreased and, in recent years, have accounted for 2%-3% of total cases, which amounts to approximately 70 cases per year [3]. In this population, prevalence decreased from 20% in the early 1990s to 10% in 2011 [4]. These changes followed the introduction of free access to syringes in pharmacies (1987), the implementation of syringe exchange programmes, low-threshold services (1990), and opiate substitution treatments (1995). However, HCV infection prevalence has decreased more modestly, from 60% in the early 2000s to 44% in 2011 (8-9% among those under 30 years of age) [4]. Transmission of HIV and HCV remains a matter of concern with the use of a growing variety of drugs and injection practices and current evidence about sharing practices and HCV seroconversion [5], especially in the context of the growing nightlife culture among youths with very diverse social backgrounds [6, 7].

Similarly to other western European countries, France has a longstanding tradition of injection drug use, mainly heroin injection [8]. In 2006, approximately 145,000 individuals in France were estimated to have used the intravenous route of administration at least once in their lifetime [9]. After a period of injection drug use decline in France in the early 2000s, recent data suggest a levelling-off of injection practices since 2008, with the overall level remaining high [10, 11], especially among the youngest users [4].

A recent European report [12] shows an upward trend in the prevalence of high-risk drug users between 2006 and 2011 in France. These results are close to the European Union average. Furthermore, ethnographic research [13] has shown the emergence of new groups of IDUs and new patterns of substance abuse. Aside from underprivileged youths, new consumers belonging to diverse social backgrounds, such as users at raves/dance parties, use a wide spectrum of substances (hallucinogens, amphetamines, synthetic products, and cocaine and its derivatives), as observed by surveillance monitoring of emerging drug use trends [14, 15]. Heroin and other opiates, including prescription opioids (morphine-sulphate and buprenorphine), are used occasionally, sometimes with other substances, for their relaxing effects. Injection is one aspect of this behaviour [16–18]. Therefore, the current harm reduction strategies, developed at a time when heroin injection was predominant, especially among injectors of low socioeconomic backgrounds, may not be reaching these new initiates to injection; these users are at high risk of exposure to HCV/HIV, which is often acquired shortly after initiation into injection [19–25].

The course of the first injection, including the circumstances and the people involved, has been shown to be particularly significant with respect to the future injection practices of new injectors [26]. Initiates are generally poorly informed regarding the techniques and risks of injection. Thus, the majority are injected or helped by experienced injectors [18, 27–33]. A recent modelling study carried out in Scotland estimated that each injector initiates approximately 0.26 individuals into injection each year [34]. Initiates are particularly vulnerable at the time of first injection because most are at the mercy of events. To date, most studies on initiation into injection drug use have been carried out among marginalised individuals who were mainly recruited through drug dependence clinics or injection drug user networks [18, 26–31, 33, 35–39]. In France, two epidemiological studies were carried out addressing the first injection in this population [30, 31]; however, none has focussed on recent initiations that took place in an era marked by changes in drug use patterns and drug-using environments.

To document the profile of new initiates, the* PrimInject* study was carried out using the Internet to reach current or former injectors and obtain descriptions of circumstances, behaviours, and potential exposure to blood-borne infections at the time of injection initiation. Launched within the context of changing trends in substance use, drug policy, and drug supply,* PrimInject* was the first study to describe new injectors and the circumstances surrounding their first injections over time [40]. The aim of this exploratory analysis is to examine injection risk behaviours and correlates at the time of initiation into injection drug use among individuals who began injection in the post-2000 era in France.

## 2. Methods

### 2.1. Population and Design

*PrimInject* used the Internet to reach young and diverse population, including individuals who had injected drugs only a few times and those who had engaged in long-term injection drug use. The electronic music scene was particularly targeted for these reasons [18, 41]. Indeed, the Internet is one of the main communication channels for participants in the electronic music scene. It is also effective in reaching small and hidden populations [42, 43]. Promotional banners using visual codes associated with the party scene invited potential participants to share their experiences with drug injection.* PrimInject* was also promoted in a wide range of harm reduction programmes and services in which the Internet was made accessible to reach drug users using low-threshold services and self-help groups. A short questionnaire was developed and pretested among drug users. Data collection took place from October 2010 to March 2011. The detailed methodology has been described elsewhere [40] and in an online methodological appendix (see Supplementary Materials available online at http://dx.doi.org/10.1155/2015/507214).

### 2.2. Questionnaire and Variables of Interest

The questionnaire covered current social status (e.g., education and employment), history of legal and illegal substance use, and the detailed circumstances surrounding the first injection. To avoid missing data, an answer was required for each question (blank responses were not allowed).

The outcomes of interest were two injection risk behaviours: (1) receptive needle sharing, defined as injection with a syringe previously used by another person at first injection, and (2) receptive sharing of other injection equipment, defined as using a cooker or cotton filter previously used by another individual.

The data on correlates pertained to several domains. One concerned participants' history of substance use before injection (use and age at first use of cannabis, ecstasy, cocaine, amphetamines, methamphetamine, ketamine, heroin, buprenorphine, methadone, other opiates, and/or hallucinogens), including heavy alcohol use (drunkenness). Early use for each substance was defined as use at 14 years of age or younger [44]. Age at first injection was also assessed and dichotomized (<18 years old and ≥18 years old).

The circumstances of first injection were documented, including age at that time, the type of substances used, year, and location (home or another private place, squat, street, outdoor location, or van/car), whether the injection took place during a party (whether in a public or private context and whether outdoors or indoors), whether the participant was alone, whether the participant injected him- or herself or was assisted, whether the first injection was planned, and whether the drug had been bought or given. The history of injection from initiation to the time of data collection was documented with two variables: the lifetime number of injections (only once, 2–10 times, 11 times, or more) and injection during the previous month (yes/no). Finally, the year of initiation was estimated by subtracting the age at the time of initiation from the age at the time of the interview. Subjects who injected earlier than 2000 were excluded from the present analysis to focus on the most recent injectors.

### 2.3. Data Analysis

Descriptive statistics were used to characterize the study sample included frequency distributions for categorical variables. To examine the association between the correlates and each outcome, univariate and multivariate logistic regression analyses were conducted. All the variables with a *p* value < 0.25 for one of the outcomes in univariate analyses, according to the Wald test, were considered for inclusion in the multivariate models. Early use of substances was not included in the multivariate logistic regressions because it was highly correlated to age at first injection.

### 2.4. Ethical Issues

Data collection was approved by the French individual data protection authority (CNIL), and safeguards on confidentiality, anonymity of responses, and the nonregistration of IP addresses were clearly stated on the home page of the survey.

## 3. Results

### 3.1. Subjects

**Figure 1:**
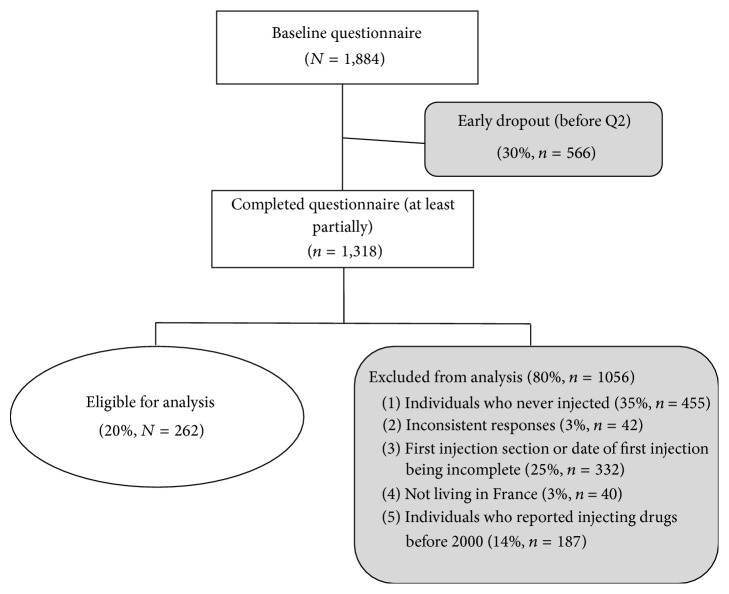
Flow chart of data cleaning and exclusion criteria from the analysis.

Among the 1,884 individuals who visited the* PrimInject* URL (http://www.shoot-premierefois.com/), 1,318 (70%) began to fill out the questionnaire. Among these individuals, 325 (25%) stopped completing the questionnaire before reaching the end of the section on first injection and were excluded from the analysis and 42 (3%) provided inconsistent answers. Most of these individuals discontinued completion or left the webpage at the very beginning of the questionnaire, in the general information section. The individuals who dropped out were younger than those who continued (mean age 27.6 versus 29.7, *p* < 0.01) and were more often students. Among the 951 respondents, 455 had never injected (48%), 40 were living abroad (4%), and seven did not report the year of their first injection. In the present study, for internal consistency, only the responses of the 262 individuals who reported injecting drugs for the first time in or after 2000 were analysed (Figure 1).

More than one-third of these respondents (37%) learned about the study through the Internet (through banners on the associations' websites) and 34% were invited to participate by outreach services (Table 1).

### 3.2. Sample Characteristics

More than two-thirds of the respondents (65%) were male and the mean age was 25 years (SD = 6) (Table 1). Only 29% of the respondents were employed, whereas 45% were unemployed and 26% were students. Regarding education, 51% had not completed high school and 26% had a college or university degree. Regarding drug use history, 64% reported early alcohol abuse and 60% early cannabis or other illegal substance use (39% only cannabis and 21% another drug). At the time of data collection, 12% reported having injected only once and 14% having injected only two to ten times in their lifetime. The majority of the sample (68%) had injected drugs at least once in the previous month.

Twenty-six percent (26%) of the respondents had experienced their first injection before the age of 18 years. Most respondents reported having injected for the first time in a private place, for example, house, apartment, or hotel (69%), and 18% during a party, whatever the context and location (8% reported having injected during a party in a private place). Initiation was planned by 36% of the sample, and 26% were alone at the time. A significant proportion of the sample reported that they had injected themselves (47%). Heroin was the drug most commonly injected at first injection (58%), followed by other opiates (15%) and cocaine and crack or freebase (15%).

### 3.3. Univariate Analyses

Among the 262 respondents, 18 did not remember whether the syringe that they used the first time had already been used (Table 2). Among the remaining 244 respondents, 8% (*n* = 20) reported that they had used a syringe already used by another person. The respondents who were female (crude odds ratio (cOR) = 2.6, *p* = 0.040), under 18 years old at the time of initiation (cOR = 4.5, *p* = 0.002), and injected by another individual (cOR = 3.1, *p* = 0.036) were more likely to report needle sharing than other participants. The association between needle sharing and initiation during a party was only marginally significant (cOR = 2.7, *p* = 0.062).

Among the 230 subjects who recalled the use of a cooker or cotton filter, 17% (*n* = 40) reported receptive sharing of this equipment. Except for one individual, all of the individuals who used a shared needle also used a cooker or cotton previously used by another person. Correlates significantly associated with sharing equipment were gender (females: cOR = 2.3, *p* = 0.020), first injection before the age of 18 (cOR = 2.9, *p* = 0.004), first injection during a party (cOR = 3.3, *p* = 0.004), not having planned to inject (cOR = 2.4, *p* = 0.031), having been injected by another person (cOR = 3.7, *p* = 0.001), and having been given the injected substance (cOR = 2.8, *p* = 0.004).

### 3.4. Multivariate Analysis

After adjustment, gender was not significantly associated with syringe or equipment sharing. Injection before the age of 18 (adjusted odds ratio (aOR) = 3.7, *p* = 0.015) and injection performed by another person (aOR = 3.1, *p* = 0.049) were positively associated with receptive syringe sharing (Table 2).

In the multivariate analysis, receptive equipment sharing was positively associated with injection before 18 years of age (aOR = 3.0, *p* = 0.011) and injection performed by another person (aOR = 3.0, *p* = 0.010) (Table 2). A borderline significant association was found with first injection during a party (aOR = 2.6, *p* = 0.053).

## 4. Discussion

The final model showed that younger age and being injected by another IDU were independently associated with increased risk in both syringe and other equipment at first injection. A nearly significant association was also found between injection at a party and equipment sharing.

To date, most epidemiological studies on the circumstances of initiation into drug injection are mainly descriptive and have been carried out among IDUs whose injection dates to the period before 2000 [26, 27, 29–31, 35–39].* PrimInject* joins the few studies investigating the independent correlates of sharing behaviours at the time of first injection [28, 45]. This study is also the first to examine initiation into drug injection using an Internet survey. Thus, a significant part of the sample was not recruited through treatment or harm reduction services, therefore allowing connection with young IDUs who were not necessarily street-entrenched or in need of services.

Our findings are consistent with the literature showing that injection equipment (cooker/cotton filter) sharing is more prevalent than needle sharing [27, 29, 30, 38, 39, 46]. The rate of sharing injection equipment among injectors of our sample is consistent with recent results from the French Ena-Caarud study among new injectors [10]. However, the definition of “equipment” is highly variable across studies and precludes valuable comparisons.

Despite a decreasing trend in drug injection thanks to harm reduction policy, injection drug use has remained relatively common in France, as shown by recent studies [4, 10], suggesting a need to study in more depth routes to entry into injection. In our study, risky injection behaviours are strongly associated with being injected by a third person, as observed in previous studies [27, 29, 47–49]. These practices might be linked to the important affective and emotional influences among couples as discussed elsewhere [32, 50, 51]. Because being injected by an experienced injector is very common (more than half of the first injections required such external help) and triples the likelihood of sharing needles and equipment, the influence of experienced users is key regarding the ability to demonstrate and practise safer use [34, 52, 53]. Interventions, such as the brief motivational intervention* Break the Cycle* [54], are designed to reduce, among people who inject drugs, injection initiation-related behaviours (e.g., speaking positively about injecting to noninjectors, injecting in front of noninjectors, and explaining or showing a noninjector how to inject) and initiation of noninjectors. The evaluation of the intervention reported that after the intervention, the participants were less likely to initiate drug users into injecting. This intervention has been recently evaluated with a peer-delivered design to increase awareness of the role of experienced users as peer educators. For example, the Toronto intervention* Change the Cycle* (CTC) included attitudes not only towards responding to young users' demand for injection but also towards teaching safer injection practices. Pilot study results suggest that CTC holds promise as a preventive intervention [55] and is currently under study in France. Our findings also support moving more broadly towards educational interventions on risks associated with drug injection, such as face-to-face educational sessions on safer injecting practices that have recently proved successful in France at reducing unsafe HIV-HCV transmission practices and injection-related complications [56].

If the unplanned injection and having been given the injected substance appear to increase the risk of sharing equipment in the univariate analyses, this association did not hold after adjustment suggesting a confounding effect. In fact, an important result of our analysis also indicates that sharing equipment was more common when injection took place during parties. The party atmosphere appeared to favour the loss of control over the situation, the group effect, and difficulty in identifying clean equipment, especially among drug users without previous injection experience [57]. A range of harm reduction programmes targeting the party scene have been launched, such as the Nevershare Syringe, with plungers in a range of colours to prevent accidental sharing [58]. In France, more specific harm reduction programmes targeting the recreational use of drugs in the techno scene have also included injection and prevention of sharing in their portfolio (http://www.technoplus.org/). The* PrimInject* findings support the renewed development of programmes targeting youth involved in the party scene, where multiple illegal substances are widely available and there is a significant chance of moving to injection practices.

Additionally, unlike previous results that indicated an association between cocaine injection and higher drug use practices [59–62], the* PrimInject* results found that, at the first injection, the use of cocaine and other stimulants did not increase the sharing of either syringes or equipment. However, early initiation into injection (under the age of 18) was correlated with needle and equipment sharing at the time of first injection. Early injection was strongly correlated with early experience of illegal drug use (*p* < 0.001): among the respondents who injected before the age of 18, 45% had used an illicit drug other than cannabis at the age of 14 or younger (versus 12% of the respondents who initiated injection at the age of 18 or older). Other individual factors were not documented to assess the various dimensions of vulnerability associated with the early onset of at-risk behaviours, such as poor family relationships, family history of alcohol and drug abuse, childhood trauma, and early school dropout [63–66]. Initiation into drug use, including injection initiation in teenage years, puts young people at increased risk of drug-related harm and calls for an update of harm reduction services to address their specific needs [67]. These results reinforce the need for early identification, referral, and intervention with young people at risk, such as the clinics for young drug users launched in France in 2004. Within this framework, a comprehensive programme for preventing a large range of addictions (e.g., tobacco, alcohol, cannabis, and video games) includes information and communication campaign and outpatient clinics adapted to young drug users and their families [68]. Early interventions, such as* Screening, Brief Intervention*, and* Referral to Treatment* (SBIRT), enable addressing high-risk drug users more effectively and help reduce harm associated with drug abuse, particularly among those who do not seek help [69]. If this public health approach has proved successful among adults, it must be tailored to the changing and evolving needs of specific young target groups and new drug trends [70]. For this purpose and because of challenges posed by changing consumption patterns among young people in the fight against infectious diseases, such as HIV and HCV, there is an urgent need to introduce new forms of intervention and services based on information and communication technologies and targeting very young drug users.

### 4.1. Strengths and Weaknesses

While prior studies on the transition to injection were mostly based on samples recruited in dedicated drug services or using targeted sampling, street outreach, or chain referral methods among mostly marginalised users [26–29, 31, 35–39],* PrimInject* reached a diverse population with respect to social status, age, past and present drug use patterns, frequency of drug use, and current behaviour and carrier in injection [40]. The study has been restricted to young people (mean age 25 years) who began injection in recent years (2000–2010) and captures a little known population that could not yet have had a long career in injection. The purpose of the study was to portray the entire spectrum of people who currently inject drugs, including those individuals with a very short history of drug injection, who represent a quarter of the sample, with 12% having injected only once and 14% two to ten times in their lifetime. These drug users are exposed to transmission of blood-borne infections, mainly HCV, in cases of unsafe practices.

Recall bias cannot be excluded, especially among those from the most remote period of initiation. Desirability bias cannot be ruled out; however, compared with more conventional modes of data collection, the anonymity of the web administration could increase the level of reporting of sensitive information and its accuracy [71, 72].

Conversely, recent injectors are more likely to use the Internet and to attend social events or services where the* PrimInject* information was promoted. This survey mode and its effort to reach electronic scene users may have biased the recruitment in favour of the least marginalised injectors. However, a sizeable proportion (approximately one-third) of the sample was recruited through outreach teams outside the Internet channel. Furthermore, the factors associated with sharing outcomes were not significantly associated with how participants reached the* PrimInject* website, either through outreach programmes or while surfing the Internet on their own. In such a hard-to-reach population, the* PrimInject* study does not claim to be statistically representative; rather, it covers a spectrum of users broader than that usually investigated [40]. Given the consistency of the* PrimInject* findings with new drug use trends in France, as regards the age of new initiates to injection and persistence of sharing equipment practices [40], the* PrimInject* results might be considered to reflect current at-risk practices and their determinants that favour the ongoing transmission of HCV.

## 5. Conclusions

The context of new patterns of drug use and emerging new consumer profiles among young people presents new challenges for harm reduction among young people. Initiation into drug use persists, and it carries potential risk for extremely diverse groups. This research identifies risk contexts during the first injection and encourages taking them into account in innovative (outreach, peer, and online) harm reduction programmes addressed both to people who inject occasionally during parties and festivals and to those who will move towards long-lasting drug use injection trajectories. In this context, the education of current injectors to protect both themselves and those they might initiate into injection is critically important. This diversification of harm reduction programmes in combination with efforts to improve access to highly effective HCV treatment could make further progress in reducing HCV prevalence and incidence.

## Supplementary Material

This appendix presents the structure, hypotheses, and methodology used to reach a socially diverse population of young people concerned with injection, as well as the design, the strategy and tools used to promote the Internet PrimInject study.

## Figures and Tables

**Table 1:** Characteristics of the sample (*N* = 262): numbers and percentages.

	*N*	%
Recruitment		
Internet	97	37.0
Outreach services	89	34.0
Drug clinics	36	13.7
Newspapers	10	3.8
Pharmacies	6	2.3
Other	24	9.2
Gender		
Male	171	65.3
Female	91	34.7
Occupation at time of data collection		
Employed	76	29.0
Unemployed	119	45.4
Student	67	25.6
Education		
<High school graduation	132	50.8
High school graduation	60	23.1
>High school graduation	68	26.1
Early substance use		
Drunkenness at the age of 14 or younger	168	64.1
Only cannabis at the age of 14 or younger	98	38.7
Other illegal drug use at the age of 14 or younger	52	20.6
Age under 18 at first injection	68	26.0
Location		
In a private location	179	68.6
At a party	47	17.9
Circumstances		
Injection planned (yes)	94	35.9
Injection while being alone	68	26.0
Injected by another person	138	52.7
Drug given (versus bought)	83	31.7
Substance first injected		
Heroin	152	58.0
Cocaine, freebase, and crack	39	14.9
Other opiates	39	14.9
Other drugs	32	12.2
Injected in the last 30 days prior to data collection	158	68.4
Lifetime number of injections		
One	31	11.8
Two to ten	37	14.1
More than ten	194	74.1

**Table 2:** Correlates of risk behaviours at time of first injection: crude odds ratios (cOR), adjusted odds ratios (aOR), and 95% confidence intervals (CI).

	Receptive syringe sharing (*N* = 244)						Receptive cooker or cotton sharing (*N* = 230)					
	cOR	95% CI	*p* value	aOR	95% CI	*p* value	cOR	95% CI	*p* value	aOR	95% CI	*p* value
Female (ref = male)	2.6^*∗*^	[1.0–6.6]	0.040	1.6	[0.6–4.5]	0.387	2.3^*∗*^	[1.1–4.6]	0.020	2.0	[0.9–4.4]	0.095
Current injector at time of data collection (injection in the last 30 days) (ref = no)	1.7	[0.5–5.3]	0.373				0.8	[0.4–1.6]	0.478			

*Characteristics and circumstances of first injection*												
Period 2000–2005 (ref = 2006–2010)	1.3	[0.5–3.2]	0.617				1.1	[0.5–2.1]	0.852			
Injection before the age of 18 (ref = no)	4.5^*∗∗*^	[1.8–11.4]	0.002	3.7^*∗*^	[1.3–10.6]	0.015	2.9^*∗∗*^	[1.4–5.9]	0.004	3.0^*∗*^	[1.3–7.0]	0.011
Injection in a private location (ref = other places (e.g., outdoors and car))	0.5	[0.2–1.4]	0.187	0.7	[0.2–1.9]	0.460	0.6	[0.3–1.2]	0.178	0.9	[0.4–2.1]	0.839
Injection during a party (whatever the context) (ref = no)	2.7	[1.0–7.5]	0.062	1.7	[0.5–5.6]	0.404	3.3^*∗∗*^	[1.5–7.4]	0.004	2.6	[1.0–6.8]	0.053
Injection not planned (ref = planned)	1.1	[0.4–2.8]	0.855	0.9	[0.3–2.7]	0.898	2.4^*∗*^	[1.1–5.3]	0.031	2.2	[0.9–5.6]	0.088
Injected by another person (ref = no)	3.1^*∗*^	[1.1–8.7]	0.036	3.1^*∗*^	[1.0–9.9]	0.049	3.7^*∗∗*^	[1.7–8.0]	0.001	3.0^*∗*^	[1.3–7.1]	0.010
Drug given (ref = bought)	1.3	[0.5–3.5]	0.545	0.9	[0.3–2.7]	0.834	2.8^*∗∗*^	[1.4–5.7]	0.004	2.0	[0.9–4.4]	0.088
Drug injected (ref = heroin)												
Cocaine, freebase, and crack	0.8	[0.2–3.1]	0.831				1.0	[0.4–2.7]	0.986			
Other opiates	0.8	[0.2–3.1]					1.1	[0.4–2.8]				
Other drugs	0.4	[0.0–3.0]					1.2	[0.4–3.6]				

*p* values of Wald tests for logistic regressions. ^*∗∗*^ *p* < 0.01; ^*∗*^ *p* < 0.05.
