# Supplementary material for: Risk Factors Associated with Unsafe Injection Practices at the First Injection Episode among Intravenous Drug Users in France: Results from PrimInject, an Internet Survey
Source: J Addict. 2015 Oct 5;2015:507214. doi: 10.1155/2015/507214 (PMC4609460; doi:10.1155/2015/507214)
Supplement: Supplementary file 1 — This appendix presents the structure, hypotheses, and methodology used to reach a socially diverse population of young people concerned with injection, as well as the design, the strategy and tools used to promote the Internet PrimInject study. [file 507214.f1.pdf]

Methodological appendix :

An advisory committee including professionals working in outreach programs,members of drug user organizations, and health administrations was set up to supervise the design of, promotion of, and tools used in the PrimInject study. Because the objective was to reach a socially diverse population of young people who recently started to inject drugs or who administered injections to themselves even once or a few times, the electronic music scene was especially considered. Indeed, fieldworkers providing harm reduction services during electronic festivals are used to distribute syringes and injection equipment to festival participants and have reported an emerging use of injection in that population (Girard and Boscher, 2009).This population, which is young, is not disadvantaged, and uses a wide variety of drugs during festivals, was targeted by using electronic music channels. The Internet is one of the main communication channel for youths and individuals participating in the electronic music scene and is considered effective to reach this small and hiddenpopulation (Velter, 2011; Frippiat and Marquis, 2010), especially young drug users, and to capture ex-drug users or individuals who had only had sporadic experiences of drug injection. Use of the Internet also limits the selection effects of treatment or harm reduction services that often overrepresent the most marginalized population or the most severe substance use profiles.The promotion messages invited people to share their first injection experienceby using an explicit URL : www.shoot-premierefois.com. Various banners and prints using the party scene visual codes were first made available on selected websites and then through a large range of harm reduction programs and services (e.g., GPs, drug dependence clinics, community pharmacies, syringe exchange programs, and spaces for youth intervention) throughout the study period. Specific attention was given to outreach services, many of which were contacted individually to provide information about the study objectives and assurance of confidentiality. Some of these services provided an Internet option for individuals to fill out the questionnaire. Data collection took place from October 2010 to March 2011. Anyone could fill out the questionnaire, regardless of whether they had injected. Respondents who had never injected were directed toward a short version of the questionnaire that explored their attitudes and opinions of drug injection. There were no lower or upper age limits for opening the questionnaire. However, respondents under 15 years of age were redirected toward the end of the questionnaire after they documented their age.
